# Elevated transaminases as a predictor of coma in a patient with anorexia nervosa: a case report and review of the literature

**DOI:** 10.1186/1752-1947-4-307

**Published:** 2010-09-17

**Authors:** Shuhei Yoshida, Masahiko Shimada, Miroslaw Kornek, Seong-Jun Kim, Katsunosuke Shimada, Detlef Schuppan

**Affiliations:** 1 Department of Gastroenterology, Internal Medicine, TMG Asakadai Central General Hospital, Saitama 351-8551, Japan; 2 Division of Gastroenterology and Hepatology, Beth Israel Deaconess Medical Center, Harvard Medical School, Boston, MA 02115, USA; 3 Medical Research Unit, Four Studies Ltd., Saitama 362-0073, Japan

## Abstract

**Introduction:**

Liver injury is a frequent complication associated with anorexia nervosa, and steatosis of the liver is thought to be the major underlying pathology. However, acute hepatic failure with transaminase levels over 1000 IU/mL and deep coma are very rare complications and the mechanism of pathogenesis is largely unknown.

**Case presentation:**

A 37-year-old Japanese woman showed features of acute liver failure and hepatic coma which were not associated with hypoglycemia or hyper-ammonemia. Our patient's consciousness was significantly improved with the recovery of liver function and normalization of transaminase levels after administration of nutritional support.

**Conclusions:**

Our case report demonstrates that transaminase levels had an inverse relationship with the consciousness of our patient, although the pathogenesis of coma remains largely unknown. This indicates that transaminase levels can be one of the key predictors of impending coma in patients with anorexia nervosa. Therefore, frequent monitoring of transaminase levels combined with rigorous treatment of the underlying nutritional deficiency and psychiatric disorder are necessary to prevent this severe complication.

## Introduction

Anorexia nervosa (AN) is a difficult-to-treat psychosomatic disease. Mild liver injury is regularly detected as a complication of AN [1-5]. Although severe acute liver injury has been previously described in a patient with AN, the underlying pathogenetic mechanisms remain largely unclear. Furthermore, only a few cases of AN with deep coma have been reported, mostly due to hypoglycemic coma [6-9].

Our case report describes a patient with AN who rapidly developed deep coma associated with acute liver failure, which was rapidly improved by initiation of total parenteral nutrition (TPN) and enteral feedings via a nasogastric (NG) tube.

## Case presentation

Our patient was a 37-year-old Japanese woman with a 12-year history of AN. She had been hospitalized frequently in the gastrointestinal unit for nutritional treatment, but she continuously rejected hospitalization in a psychiatric unit probably due to her denial of the illness, despite her frequent, self-induced vomiting. She had been admitted to the hospital three times previously because of general fatigue combined with acute liver failure. At these admissions, her Glasgow coma scale (GCS) was in the normal range of 12 to 15. She was strongly recommended to consult a psychiatrist, but turned down the advice because of denial of her AN. Therefore, she was only prescribed ursodeoxycholic acid, multivitamin, and an enteral nutritional supplement. She had never previously received any psychotherapy. Furthermore, she had no history of alcohol abuse, anti-depressant drug intake, narcotic drug abuse or suicide attempt.

On the three prior admissions, physical examination had revealed severe emaciation, with a weight of 29.0 kg and a height of 1.52 m (body mass index (BMI) = 12.6). Her body temperature was below 36°C, her blood pressure was around 85/50 mm/Hg, with a regular heart rate of around 80 beats per minute.

At the time of her fourth admission, she was in a deep coma with a GCS score of 3. Arterial blood gas analysis revealed an arterial oxygen concentration of 97% in room air. The electrocardiogram showed sinus rhythm and a heart rate of 88 beats per minute. She had a weak, but positive papillary response without papillary mydriasis or miosis. Her body temperature was 35.6°C. There were no signs of respiratory or cardiac disease. Her blood sugar level was 68 at the time of admission, in the range of her usual level of 50 to 70. Computed tomography (CT), magnetic resonance imaging, and magnetic resonance angiography of the head showed no abnormality.

Aspartate aminotransferase was 3194 IU/L (reference range, 7 to 38 IU/L); alanine aminotransferase, 3540 IU/L (4 to 44 IU/L); alkaline phosphatase, 2388 IU/L (100 to 320 IU/L); γ-glutamyl transpeptidase, 342 IU/L (2 to 40 IU/L); NH_3_, 51 μg/dL (40 to 80 μg/dL). The ratio of branched-chain amino acids versus aromatic amino acids (BCAA/AAA) was 3.8 (2.5 to 3.5); albumin was 3.6 g/dL (3.8 to 5.3 g/dL); total bilirubin, 1.7 mg/dL (0.2 to 1.0 mg/dL); total cholesterol, 117 mg/dL (130 to 220 mg/dL); prothrombin activity, 49.8% (80 to 120%); hepaplastin test, 50.1% (70 to 130%); Type IV 7S-collagen, 4.9 ng/mL (< 6.0 ng/mL); HbA1_c_, 4.0% (4.3 to 5.8%); blood urea nitrogen, 23.6 mg/dL (8.0 to 20.0 mg/dL); creatinine, 0.69 mg/dL (0.3 to 0.8 mg/dL); white blood cells, 4070/mL (3800 to 9300/mL); hemoglobin, 12.1 g/dL (11.5 to 15.0 g/dL); hematocrit, 34.5% (33.5 to 44.5%); Fe, 123 μg/dL (48 to 154 μg/dL); Cu, 78 μg/dL (66 to 130 μg/dL); Zn, 92 μg/dL (59 to 135 μg/dL); platelet count, 12.7 × 10^4^/μL (13 to 37 × 10^4^/μL); and total protein, 5.0 g/dL (6.5 to 8.2 g/dL). Anti-nuclear and anti-mitochondrial antibodies were negative. Serologic tests for hepatotropic viruses (hepatitis A, B, and C viruses, cytomegalovirus, and Epstein-Barr virus) and the urinary toxicology screen (alcohol, cannabis, cocaine, paracetamol, amphetamines, benzodiazepines, methadone, opiates) were negative. Ultrasound showed a mild fatty liver, but the CT score (Hounsfield units) of the liver was slightly higher than that of the spleen (data not shown).

**Figure 1:**
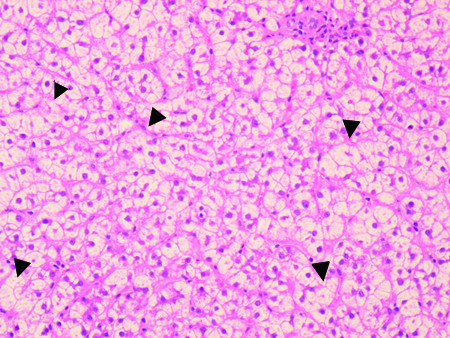
**Hematoxylin-eosin staining of liver biopsy specimen of the patient with anorexia nervosa**. Diffuse macrovesicular steatosis as well as numerous ballooning hepatocytes. Necroinflammatory changes representing acidophilic bodies and spotty necrosis (arrowheads).

The NH_3 _and BCAA/AAA levels remained normal during our patient's coma and afterward, and the blood sugar remained close to her usual level (Table 1). TPN and enteral tube feeding were administered on the day of admission. Her consciousness gradually normalized at day 10, which was paralleled with an improvement of her severe liver dysfunction (Table 1). Comparing the broad spectrum of laboratory clinical parameters with her GCS level, only serum transaminases showed a strong inverse correlation. Of note, blood sugar, plasma NH_3_, and the BCAA/AAA ratio were not correlated with her consciousness. A liver biopsy was performed after the recovery of her liver function at day 14. Ballooning of hepatocytes, necroinflammatory changes, and macrovesicular steatosis were observed in hematoxylin-eosin-stained sections (Figure 1), but both iron and copper staining were negative (data not shown). No etiology of the deep coma, other than acute malnutrition-induced liver injury, was detected.

**Table 1:** Laboratory data at admission and during hospitalization.

	Day 1	Day 2	Day 3	Day 4	Day 5	Day 7	Day 10	Day 14	Day 19	Day 25	Day 32
AST (IU/L) (7-38)	3194	4880	2556	1614	1567	1021	807	455	138	86	45
ALT (IU/L) (4-44)	3540	5408	4056	3672	2440	1958	1492	859	434	137	70
ALP (IU/L) (100-320)	2388	3282	2872	2732	1948	1649	1080	741	651	524	482
T-BIL (mg/dL) (0.2-1.0)	1.7	2.3	2.7	2.2	2.5	2.1	2.0	1.4	1.5	1.2	0.9
ALB (g/dL) (3.8-5.3)	3.1	3.6	3.3	3.1	3.4	3.3	3.3	3.5	3.2	2.8	2.9
NH_3 _(μg/dL) (40-80)	51	28	69	33	36	73	88	90	77	80	80
PTA (%) (80-120)	49.8		44.6		48.3	54.5		67.9		79.4	
BS (mg/dL) (70-160)	68	98	89	102	94	87	85	68	66	58	62
BCAA/AAA (2.5-4.5)	3.8		3.6			3.3		4.0		3.1	3.2
GCS (15)	3	3	4	5	7	10	15	15	15	15	15

## Discussion

To the best of our knowledge, this is the first report of a patient with AN presenting with deep coma associated with acute hepatitis/liver failure. AN is an eating disorder, affecting mainly young women with a distorted body image and a overwhelming desire to be slim. Minor degrees of liver injury have been reported in up to 40% of patients with AN [5]. Although the mechanism of liver injury in AN has been thought to be due to protein-calorie malnutrition of the Kwashiorkor-type with fatty changes, this has not been rigorously demonstrated, and the precise mechanism is still unknown [10]. Starvation-induced autophagy of hepatocytes [11] and enhanced starvation-induced hepatocyte oxidative stress may be a leading mechanism resulting in liver dysfunction in AN [12]. In the latter report, the CT density of the liver was higher than that of the spleen in a patient with AN and elevated transaminases, whereas liver steatosis was diagnosed in ultrasound imaging, as was found in our patient. In addition, these authors detected increased markers of oxidative stress in the liver biopsy. Again, this is compatible with our finding of numerous hepatocytes with signs of ballooning (Figure 1), which is a hallmark of oxidative stress and of hepatocyte apoptosis and autophagy in alcoholic and non-alcoholic steatosis [13]. These reports, in conjunction with our findings, strongly indicate that starvation in AN patients leads to enhanced oxidative stress, hepatocyte apoptosis, and autophagy that trigger acute liver inflammation and moderate functional liver failure. To date, only rare cases describe coma in AN patients, most of them due to hypoglycemia [6-9]. Hypoglycemia could be ruled out in our case. Interestingly, an inverse relationship was noted between the GCS and the transaminase levels (Table 1). This further supports the previously mentioned sequence in which acute starvation-induced liver injury apparently promoted the development of hepatocyte necrosis/autophagy, liver dysfunction, and deep coma in a patient with AN. However, this hypothesis does not necessarily apply to all patients with severe hepatitis, because a case of a patient with AN with clear consciousness despite highly elevated transaminase was reported [14]. The present case is different from other cases of acute or chronic or liver failure, in that the circulating type IV 7S-collagen, the BCAA/AAA ratio, and the NH_3 _level remained normal during several days of deep coma. Recently, in two patients with AN and normal transaminase levels, iatrogenic hyperammonia induced by high-protein dietary supplements was reported [15]. In our case, coma gradually disappeared with improvement of nutritional status and liver injury, but was unrelated to the NH_3 _level, usually a strong predictor of encephalopathy in acute or cirrhotic liver failure [15]. The clinical data clearly indicated that the transaminase levels had a strong inverse correlation with our patient's consciousness. These results strongly suggest that the pathogenesis of coma in classic hepatic encephalopathy differs from that in our patient with AN.

## Conclusions

Our case report of a patient with AN and high transaminase levels in a deep coma indicates that severe starvation-induced hepatocyte autophagy and apoptosis may lead to a diagnosis of acute liver failure. However, in contrast to hepatic encephalopathy, neither blood ammonia levels nor the ratio of BCAA/AAA was abnormal. We hypothesize that patients with AN and mild liver dysfunction may develop lower degrees of encephalopathy that may escape routine detection. Therefore, it is necessary to monitor transaminase levels regularly in patients with AN.

It is important to note that the severe hepatitis and encephalopathy observed in our patient were completely reversed after institution of appropriate parenteral and enteral nutrition. We hope that psychiatric therapy will remain the mainstay of treating patients with AN, preventing severe malnutrition with subsequent liver dysfunction, as was diagnosed in our patient.

## Abbreviations

ALB: albumin; AN: anorexia nervosa; AST: asparate aminotransferase; ALT: alanine aminotransferase; BCAA/AAA: branch-chain amino acid/aromatic amino acid; BS: blood sugar; GCS: Glasgow Coma Scale; NH_3_: ammonia; PTA: prothrombin activity; T-BIL: total bilirubin.

## Competing interests

The authors declare that they have no competing interests.

## Consent

Written informed consent was obtained from the patient for publication of this case report and any accompanying images. A copy of the written consent is available for review by the Editor-in-Chief of this journal.

## Authors' contributions

SY and MS contributed equally to the management of the patient and the researching for and writing of this manuscript. SY mainly wrote the manuscript. MK, SK, and KS commented on drafts and did literature searches. DS advised and wrote and revised the manuscript. All authors read and approved the final manuscript.
